# Pathogenesis and Surgical Treatment of Congenitally Corrected Transposition of the Great Arteries (ccTGA): Part III

**DOI:** 10.3390/jcm13185461

**Published:** 2024-09-14

**Authors:** Marek Zubrzycki, Rene Schramm, Angelika Costard-Jäckle, Michiel Morshuis, Jochen Grohmann, Jan F. Gummert, Maria Zubrzycka

**Affiliations:** 1 Department of Surgery for Congenital Heart Defects, Heart and Diabetes Center NRW, University Hospital, Ruhr-University Bochum Georgstr. 11, 32545 Bad Oeynhausen, Germany; mzubrzycki@hdz-nrw.de; 2 Clinic for Thoracic and Cardiovascular Surgery, Heart and Diabetes Center NRW, University Hospital, Ruhr-University Bochum, Georgstr. 11, 32545 Bad Oeynhausen, Germany; rschramm@hdz-nrw.de (R.S.); ajaeckle@hdz-nrw.de (A.C.-J.); mmorshuis@hdz-nrw.de (M.M.); jgummert@hdz-nrw.de (J.F.G.); 3 Department of Congenital Heart Disease/Pediatric Cardiology, Heart and Diabetes Center NRW, University Hospital, Ruhr-University Bochum, Georgstr. 11, 32545 Bad Oeynhausen, Germany; jgrohmann@hdz-nrw.de; 4 Department of Clinical Physiology, Faculty of Medicine, Medical University of Lodz, Mazowiecka 6/8, 92-215 Lodz, Poland

**Keywords:** congenitally corrected transposition, discordant atrioventricular connections, discordant ventriculoarterial connections, diagnosis, operation procedures

## Abstract

Congenitally corrected transposition of the great arteries (ccTGA) is an infrequent and complex congenital malformation, which accounts for approximately 0.5% of all congenital heart defects. This defect is characterized by both atrioventricular and ventriculoarterial discordance, with the right atrium connected to the morphological left ventricle (LV), ejecting blood into the pulmonary artery, while the left atrium is connected to the morphological right ventricle (RV), ejecting blood into the aorta. Due to this double discordance, the blood flow is physiologically normal. Most patients have coexisting cardiac abnormalities that require further treatment. Untreated natural course is often associated with progressive failure of the systemic right ventricle (RV), tricuspid valve (TV) regurgitation, arrhythmia, and sudden cardiac death, which occurs in approximately 50% of patients below the age of 40. Some patients do not require surgical intervention, but most undergo physiological repair leaving the right ventricle in the systemic position, anatomical surgery which restores the left ventricle as the systemic ventricle, or univentricular palliation. Various types of anatomic repair have been proposed for the correction of double discordance. They combine an atrial switch (Senning or Mustard procedure) with either an arterial switch operation (ASO) as a double-switch operation or, in the cases of relevant left ventricular outflow tract obstruction (LVOTO) and ventricular septal defect (VSD), intra-ventricular rerouting by a Rastelli procedure. More recently implemented procedures, variations of aortic root translocations such as the Nikaidoh or the half-turned truncal switch/en bloc rotation, improve left ventricular outflow tract (LVOT) geometry and supposedly prevent the recurrence of LVOTO. Anatomic repair for congenitally corrected ccTGA has been shown to enable patients to survive into adulthood.

## 1. Introduction

Congenitally corrected transposition of the great arteries (ccTGA), or double discordance, is a rare congenital malformation. Its prevalence is estimated at approximately 1/33,000 live births, accounting at the same time for 0.5% of all congenital heart diseases [1,2]. ccTGA is part of the family of conotruncal anomalies, characterized by double atrioventricular (AV) and ventriculoarterial (VA) discordance. Thus, the circulation is correct physiologically—with pulmonary venous blood being directed to the systemic circulation and systemic venous blood to the pulmonary circulation. However, the systemic ventricle is the morphological right ventricle (mRV), guarded by the tricuspid valve, whereas the subpulmonary ventricle is a morphological left ventricle (mLV) guarded by the mitral valve.

The classification of this anomaly is complex due to the wide spectrum of morphological variants and associated cardiac anomalies coexisting under the heading of ccTGA. This anatomical and morphological heterogeneity is reflected in the variable and unpredictable clinical presentations of the condition—from newborns with large VSD and arch obstruction necessitating urgent surgical intervention, to adults with no associated lesions who can live a normal life with no need for any treatment. The most common intracardiac lesions associated with ccTGA, seen in 80%–90% of all cases, are ventricular septal defect (VSD), pulmonary stenosis (PS), left atrioventricular (AV) valve (morphological tricuspid valve) regurgitation, and/or complete heart block, as well as conduction abnormalities [3].

Patients with cc-TGA and without any other associated lesions remain asymptomatic until adulthood, when they present with sRV dysfunction manifesting clinically as valvular disease, heart block, and heart failure. Although indications for surgery are usually related to these associated lesions, it is also noteworthy that even “isolated ccTGA” (i.e., ccTGA with no other associated cardiac anomalies) can become symptomatic in view of the unpredictable nature of the mRV in the systemic circulation. Progressive mRV failure and tricuspid regurgitation can present the signs and symptoms of congestive cardiac failure at any age, and the inter-relationship of primary ventricular dysfunction leading to tricuspid regurgitation or of primary TV dysfunction leading to ventricular failure is a complex issue, each leading to a vicious circle of progress. The optimal surgical strategy for ccTGA treatment depends on the specific anatomical structures as well as the prevention and management of heart failure, and requires sufficient individualized consideration. Surgical strategies include physiological correction and anatomical correction. The traditional surgical approach (physiological repair) of ccTGA attempts to restore normal physiology by repairing the associated lesions. It does not address the most serious anatomic abnormalities, mainly ventriculoarterial discordance, and results in long-term outcomes that are less than optimal. Anatomic repair was introduced with the aim to incorporate the left ventricle into the systemic circulation. Due to the excellent short-term and intermediate results of the double-switch operation and its modifications, it has become the procedure of choice for the treatment of ccTGA.

Different types of anatomic repair aiming for the correction of double discordance have been proposed. They combine an atrial stage switch (Senning or Mustard procedure) together with either the Jatene arterial switch operation (ASO) as a double-switch procedure or, in cases of significant left ventricular outflow tract obstruction (LVOTO) and ventricular septal defect (VSD), as intra-ventricular rerouting by means of a Rastelli procedure [3], as well as variants of aortic root translocations such as the Nikaidoh or the half-turned truncal switch/en bloc rotation procedures [4,5,6].

No single approach appropriate for all patients has been developed since the disease is rare and the specific anatomic and physiologic manifestations are highly variable. Most of the repairs have to be tailored individually.

The aim of this paper was to present the pathogenesis and possibilities of surgical treatment of ccTGA.

## 2. Definition and Pathogenesis of ccTGA

**Figure 1:**
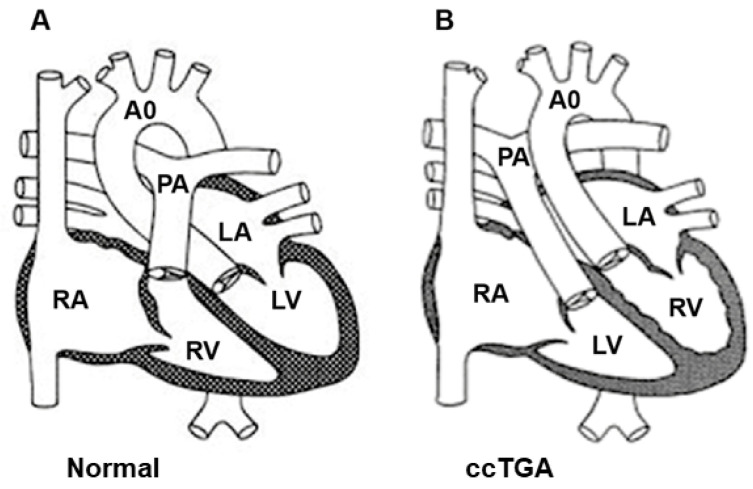
Diagrams of the normal heart (**A**) and ccTGA (**B**). In the normal heart, the pulmonary artery arises from the right ventricle, and the aorta arises from the left ventricle (RA with LV, LA with RV). In ccTGA, the right atrium is connected to the morphological LV, which ejects blood into the pulmonary artery, whereas the left atrium is connected to the morphological RV, which ejects blood into the aorta. The ventricles are inverted. RA: right atrium; RV: right ventricle; PA: pulmonary artery; LA: left atrium; LV: left ventricle. This figure was modified and reproduced with permission from Goldmuntz et al. [9].

The entity currently known as congenitally corrected transposition of the great arteries (ccTGA) was first described by an Austrian pathologist, Carl von Rokitansky, in 1875 [7]. The anomaly is also commonly referred to as levo- or L-looped transposition of the great arteries (L-TGA), double discordance, or ventricular inversion. Its essence is atrioventricular (AV) and ventriculoarterial (VA) discordance. Anomalous connections of the atria with the ventricles are accompanied by the transposition of the great arteries [8] (Figure 1).

This anomaly is very rare, accounting for approx. 0.5–1% of congenital heart defects (CHD), which translates into an incidence of 0.02/1000 live births [2,10].

The causes of abnormal embryonic development of the heart leading to the development of this anomaly are not definitively understood [2]. It is believed that as a result of disorders in the development of the primary cardiac tube, the bulboventricular part of the primary heart forms a left-sided loop instead of a right-sided loop—the normally located atria of the heart are connected to morphologically incompatible ventricles. This anomaly is accompanied by abnormal torsion of the aortopulmonary septum (transposition of the great vessels). As a result, blood flow in the cardiovascular system is not disturbed, because the anatomy of the heart, despite abnormal connections, contributes to the “correction” of circulatory physiology. Non-oxygenated blood from the systemic veins flows into the right atrium and through the mitral valve to the morphologically left ventricle (venous ventricle), which pumps blood to the pulmonary artery located posteriorly, whereas oxygenated blood from the pulmonary veins flows to the left atrium and through the tricuspid valve to the morphologically right ventricle (arterial ventricle), which pumps blood to the anteriorly located aorta. The right ventricle acts as the systemic chamber. The left-sided position of the aortic valve in relation to the pulmonary artery valve is the basis for the currently preferred term “L-transposition of large arteries”. Both large arteries run, as in D-TGA, in parallel rather than crossing as they do in the case of concordant ventriculoarterial connections [11,12,13].

This defect occurs in two forms: as an isolated defect (1–2%) without associated anomalies [14] and as a complex defect, which is associated with additional anomalies and occurs much more frequently—in approx. 80–90% of cases [12,13,15,16].

## 3. Anatomical Variants of ccTGA

Richard von Praagh distinguished several anatomical variants of this defect depending on the segmental structure of the heart [17]. The simplest form of ccTGA presents as an isolated combination of atrioventricular and ventriculoarterial discordance with no other structural abnormalities in SLL configuration.

1. Cardiotype {S,L,L}—normal (situs solitus) atrial position and ventricular inversion (ventricular L-loop), i.e., the morphologically left atrium (LA) and right atrium (RA) are located on the left and right sides, respectively. The morphologically right ventricle is on the left and the left ventricle is on the right. The great arteries are transposed (ventriculoarterial discordance), the aorta is connected to the anatomically right ventricle located on the left, and the pulmonary artery is connected to the right-sided anatomically left ventricle; the great vessels are L-malposed with the aorta anteriorly and to the left of the main pulmonary artery.

The right atrium is thus connected by the mitral valve to the morphologically left ventricle displaced to the right and towards the front, which connects through the outflow tract with the pulmonary artery that is slightly shifted backwards. The left atrium connects through the tricuspid valve to the right ventricle, which connects to the left-located aortic valve and the ascending aorta via the right ventricular outflow tract located slightly to the front. The ventricular outflow tracts in the corrected transposition are parallel, and the interventricular septum is located in the anteroposterior position.

The anatomy of the coronary arteries presents a mirror image of their normal position. In ccTGA, the coronary arteries are inverted, arise from the anterior and posteriori aortic sinuses, and follow their respective ventricles. The right-sided coronary artery reflects the anatomy of the left coronary artery and gives rise to the anterior descending and circumflex arteries, which run in the atrioventricular groove behind the mitral valve located on the right. The left-sided coronary artery runs to the atrioventricular sulcus between the left atrium and the right ventricle and branches similarly to the normal right coronary artery.

The blood supply to the systemic RV is provided only by the left-sided (morphologically right) coronary artery, which can lead to inadequate perfusion of the hypertrophied ventricular mass and the RV function deteriorating with age. The anomaly of the conduction pathway involves the anterior position of the atrioventricular node at the junction of the right-sided mitral valve annulus and the limbus of the septum secundum [18].

2. Cardiotype {S, L, D}—corrected transposition with a normal visceroatrial relationship (situs solitus) and ventricular L-loop, i.e., atrioventricular and ventriculoarterial discordance; there is a D-relation of the large vessels, i.e., the aorta lies on the right side in relation to the pulmonary artery trunk.

3. Cardiotype {I, D, D}—physiologically corrected position of the great vessels in complete visceral inversion (situs inversus) (10% of cases). This is a mirror image of the {S, L, L} cardiotype. In {I, D, D} ccTGA, the aorta is located to the right and anteriorly in relation to the pulmonary trunk, while in {S, L, L} ccTGA it is located to the left and anteriorly in relation to the posterior pulmonary trunk. In situs inversus, the morphologically right atrium is located on the left and the left atrium on the right. The apex of the heart is directed to the right, which should be differentiated from isolated dextrocardia in the correct position of the viscera.

## 4. Anomalies Associated with ccTGA

In the isolated form, the defect may remain asymptomatic for a long time, but its course may be associated with frequent arrhythmias and conduction disorders requiring intervention, as well as systemic ventricular insufficiency [8].

The natural history of ccTGA is highly variable and depends largely on associated cardiac anomalies. Most cases of congenitally corrected transposition have one or more coexisting malformations. The most frequent concomitant additional anomalies include ventricular septal defect (VSD) (75–80%), valvular or subvalvular stenosis of the pulmonary artery (pulmonary stenosis (PS) (40–53%), morphological tricuspid regurgitation (TR), and structural anomalies of the tricuspid valve (53–94%) [12]. Additionally, patients with ccTGA may have abnormalities in the structure of the atrioventricular node, which leads to conduction disorders. In these patients, high AV-block rates of 30–50% have been reported by the age of 50 years, with an annual risk of developing a de novo AV block of ~2% [19,20,21]. Patients with AV discordance are at risk of developing a complete AV block throughout their lives. Tricuspid regurgitation (pulmonary vascular congestion) and dysfunction of the systemic ventricle become manifest in such patients, most frequently in the fourth decade of life. The risk of atrial tachyarrhythmias, which can trigger pulmonary edema, is increasing.

Ventricular septal defect (VSD) of conotruncal type is present in approx. 70% to 80% of patients with some malalignment and occasional inlet extension. It is perimembranous in nature, usually extensive, may be located under the arterial valves (subpulmonary or subaortic), and is limited by the semilunar valve—the left-sided tricuspid valve. The defect may be accompanied by straddling and/or overriding of the tricuspid or mitral valve. In such cases, the VSD can extend into the inflow part of the interventricular septum. Atrioventricular defect (atrioventricular canal type) with subpulmonary stenosis in the left ventricular outflow tract is rarely reported [8].

Valvular or subvalvular stenosis of the pulmonary artery (pulmonary stenosis) coexists with ccTGA in approx. 40% of cases and is usually associated with VSD. It is most often caused by fibromuscular tissue connecting to the membranous part of the ventricular septum, tricuspid valve, or pulmonary valve [22]. Pulmonary atresia, representing the extreme form of pulmonary obstruction, can also be observed in the setting of congenitally corrected transposition [2].

## 5. Tricuspid Valve Regurgitation (TR)

Tricuspid valve regurgitation (TR) is a key prognostic overall survival determinant in ccTGA patients. Progressive TR occurs with age and is associated with worsening RV function [3,8]. Long-term survival (20 years) has been observed in 47% of patients with at least moderate TR and 93% of those without TR [23]. Studies have also demonstrated that 94% of patients with ccTGA suffered from intrinsic tricuspid valve abnormalities [13].

Three distinct types of TR in sRV patients: annular dilatation, valvular prolapse, and valve tethering were identified by Szymanski et al. [24]. TR due to valve tethering is associated with a considerably greater degree of sRV dysfunction than annular dilatation or valve prolapse [24].

The development of TR results from the widening of the right ventricle and the subsequent dilatation of the tricuspid valve ring, which begins to resemble a saddle. Additionally, the change in the geometry of the anatomically loaded right ventricle in terms of pressure and volume (as a result of the valve regurgitation) results in the interventricular septum becoming flat or even concave, thus changing the spatial arrangement of the subvalvular apparatus and reducing the coaptation of the leaflets.

Apical displacement of the septal and mural leaflets occurs [25].

It is also intensified by reduced blood supply, anatomically enlarging the right ventricle and thus contributing to the increasing dilation of the tricuspid ring. The consequence of this phenomenon is an increasing impairment of the anatomically right systemic ventricle function. Interestingly, this relationship is neither constant nor linear, as it has been observed that severe tricuspid regurgitation does not occur in all patients with right ventricular insufficiency.

## 6. Systemic Right Ventricle (sRV) Dysfunction

Systemic right ventricle (sRV) failure is mainly a result of its inability to adapt to high pressures, leading to ventricular hypertrophy, and later to dilatation, dysfunction, and eventually heart failure (HF) with significant long-term morbidity and mortality [16]. The dysfunction of the sRV can develop at any stage, but it is almost uniformly present in the population of older patients, which indicates that the anatomic RV is unable to sustain the systemic circulation in the long run [26]. Heart failure due to severe TR and sRV dysfunction is the most common presentation in adult patients diagnosed with ccTGA. Approximately 30% of patients with simple ccTGA and 60% of those with associated lesions develop cardiac failure by 45 years of age [16].

The pathophysiology of systemic right ventricular dysfunction is multifactorial and heterogeneous and includes a long-term increased afterload of the sRV, which leads to the remodeling of the myocardium that is not adapted to function in a high-pressure system. The wall of the ventricle thickens and changes its shape (dilation). At the same time, changes in the architecture of muscular fibers are observed—from longitudinal fibers predominating in the right ventricle towards fibers with a circular course (like in the left ventricle). In patients with ccTGA, fibrosis of the sRV muscle may also occur. These complex compensatory mechanisms and pathological myocardial hypertrophy together with microcirculation disorders (myocardial vascularization originates from only one—the right—coronary artery) lead to sRV dysfunction and the development of congestive heart failure in long-term follow-up [16,26].

Various imaging modalities have been used in clinical practice to evaluate the systemic RV, but echocardiography is the first-line diagnostic modality, providing information on the size and systolic function of the sRV.

Analogously to the LV, the RV ejection fraction (RVEF), the global longitudinal strain (GLS) of the right and left ventricles (RV/LV), the assessment of echocardiography-derived myocardial work (MW) analysis, and the atrial strain are considered to be the markers of RV function [27].

Recently, Fusco et al. investigated the value of echocardiographic myocardial function analysis in assessing RV systemic function in ccTGA. MW was measured from the speckle tracking analysis of global longitudinal strain (GLS) using non-invasively estimated ventricular pressure to obtain LV pressure–strain loops, thus incorporating LV afterload to provide a more accurate evaluation of LV efficiency, regardless of ventricular afterload conditions. In their cohort, MW revealed biventricular impairment, in particular for non-systemic LV, in spite of normal GLS values. These are the first data on the use of MW analysis in patients with sRV [27].

Yoshida et al. compared measures of ventricular function in physiologically repaired sRVs and anatomically repaired sLVs in ccTGA and found an advantage of anatomical repair over physiological repair with respect to long-term systemic ventricular function and NYHA classification [28].

Cardiac magnetic resonance (CMR) is useful in identifying subclinical sRVd and it is considered the gold standard to measure and assess sRV volume and function, sRV fibrosis, and the potency or leakage of atrial baffles. The estimation of RV GLS, measured with STE, in combination with RVEF%, as well as RV volumes and mass determined with CMR, can effectively identify patients at the highest risk of advanced HF or death [2,3,19].

Despite the fact that the treatment regimens of patients with left ventricular failure are well established, the clinical management of patients with systemic right ventricular dysfunction is still a challenge. The use of cardiac resynchronization therapy is increasing, whereas pharmacological therapy is largely empirical. The options applicable in end-stage heart failure when other management strategies have been exhausted include mechanical assist device and heart transplantation.

Due to the considerable rarity of the disease, most data on sRV function in ccTGA are derived from small groups of patients or case reports. In addition, some of the data come from sRV studies, in which conclusions were drawn based on a joint analysis of the populations of patients with D-TGA after atrial correction and patients with ccTGA (usually a much smaller population). Therefore, any information on the assessment of sRV function in adults with ccTGA and disease-modulating factors can support clinicians in developing new diagnostic algorithms, making recommendations, and making clinical decisions.

## 7. Conduction System and Conduction Disorders in ccTGA

A characteristic anatomical feature of the ccTGA is the specific structure and topography of the conduction system. The atrioventricular conduction system is abnormally positioned as a result of the malalignment of the atrial and ventricular septal structures [3,19].

The anatomical disposition of the cardiac conduction system is different in the two forms of ccTGA: those with the usual atrial arrangement (situs solitus SS-ccTGA) versus those with a mirror-imaged arrangement of atrial appendages (situs inversus SI-ccTGA) [19,29,30].

Hearts with the SS-ccTGA usual atrial arrangement variant have a grossly abnormal disposition of the atrioventricular conduction system [19,29,30,31]. In this relation, the bundle of His does not connect to the atrioventricular node located within the Koch’s triangle. It departs from the additional atrioventricular node located near the junction of the atrial septal tissue with the mitral valve ring, in the commissure region between the anterior and posterior mitral valve leaflets. The bundle of His, after passing the fibrous triangle forming a fibrous continuity between the mitral valve and the valve of the pulmonary trunk, traverses the anterior wall of the left ventricle outflow tract and runs under the right septal leaflet of the pulmonary valve; then, its course is subendocardial along the left surface of the interventricular septum.

**Figure 2:**
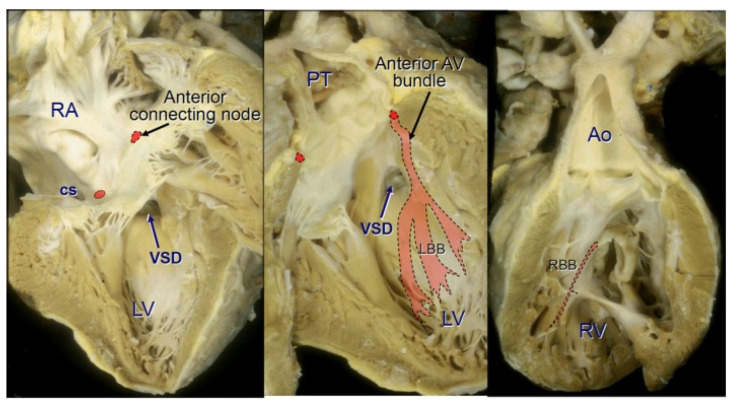
Disposition of cardiac conduction system in ccTGA. Ao indicates the aorta; AV, atrioventricular; cs, coronary sinus; LBB: left bundle branch; LV: left ventricle; PT: pulmonary trunk; RA: right atrium; RBB: right bundle branch; RV: right ventricle; VSD: ventricular septal defect. This figure was taken from the article of Baruteau et al. [19] distributed under the terms of the Creative Commons Attribution License (CC BY).

Reaching the ventricular septum, the bundle turns inferiorly to descend anteriorly along the septum at some distance before it branches to give rise to the left bundle branch on the right side and the right bundle branch on the left side (Figure 2).

By contrast, in the mirror-imaged atrial arrangement, the sinus node is situated in the left-sided atrium in its usual position in relation to the terminal crest and the entrance of the superior caval vein. Such hearts are characterized by a better alignment of the atrial and ventricular septa, allowing a regularly located atrioventricular node at the apex of the Koch’s triangle, to continue in normal fashion to an atrioventricular bundle passing in a posteroinferior relationship to the margin of a VSD if there is one [32]. The bundle branches descend in the usual manner—a cord-like right bundle branch to the right side of the septum and a fan-like left bundle branch to the left side. After ramification, the bundle branches may continue as an anterior bundle that ends blindly, without making contact with another, anteriorly located, atrioventricular node [19].

If a VSD is present, then the bundle runs close to the pulmonary valve, around the LVOT and on the left side, along the anterosuperior margin of the defect (in contrast to a normal heart with cardiotype {S, D, S}—where it runs along the posteroinferior margin of the VSD). In some patients, an additional, posterior atrioventricular node has been described, from which the bundle running along the posteroinferior margin of the VSD departs. In these cases, the VSD may be surrounded by a bundle of conductive tissue. In {l, D, D} ccTGA, the atrioventricular bundle departs from the posterior atrioventricular node (within the Koch’s triangle) and runs along the posteroinferior margin of the VSD (if present) on the side of the morphologically left ventricle [31].

Due to anomalies of the conductive system, arrhythmia and conduction disorders are common symptoms in this group of defects. Complete atrioventricular block occurs in 12–33% of cases and is more common in patients with a continuous ventricular septum. Supraventricular tachycardias and premature ventricular excitation syndrome may occur. Spontaneous (1–2% of the patient/year) atrioventricular block, which can occur at any time from the neonatal period to old age, is observed with high frequency. Atrioventricular (AV) block as a result of TV and VSD surgery has been described with a frequency of up to 30% and is associated with both the structure of the conductive system and the process of fibrosis, and even calcification within the junction between the atrioventricular node and the atrioventricular bundle, and increases with age [8,19,21,33].

## 8. Symptomatology of ccTGA

The symptomatology of the disease is related to its associated malformations. In the cases without concomitant defects, the clinical course of ccTGA is asymptomatic early in life, and often even for several decades. In most female patients, ccTGA is diagnosed after delivery, although antenatal diagnosis has been reported [34]. It is noteworthy that this defect is still diagnosed in all age groups, from the neonatal age through adulthood to 80 years of age [35,36]. Some patients can live a full life without any problems, the entity being discovered accidentally during autopsy. Cyanosis and dyspnea are the commonly presented symptoms. They are a result of failing RV or tricuspid valve functioning at systemic pressure or associated anomalies such as LVOTO and VSD [35,37]. Depending on the predominant lesion, they may present with heart failure (secondary to pulmonary overcirculation). However, heart failure will develop earlier in life if there is a hemodynamically significant VSD, evidenced in children by easy fatigability, poor weight gain, feeding intolerance, etc. Bradycardia with symptoms of congestive circulatory failure due to atrioventricular block may occur spontaneously, which may manifest as syncopes. The symptoms of circulatory failure usually occur in the fifth–sixth decades and are associated with increasing tricuspid valve and sRV insufficiency [35].

## 9. Prenatal Diagnosis of ccTGA

The diagnosis of ccTGA can be established easily during fetal life by an experienced sonographer. Suspicious pregnancies are usually referred to the fetal echosonographer if there is a positive family history of congenital heart disease, or when fetal screening has revealed other congenital heart defects.

A recent study demonstrated that there are several features which should alert the sonographer to the possibility of ccTGA [38]. The reversed off-setting of the atrioventricular valves was found in three-quarters of fetuses, while the moderator band was identified in the left-sided or posterior ventricle in nearly nine-tenths. The diagnosis is more difficult in the case of isolated corrected transposition, since there are no other heart defects prompting the sonographer to carry out a detailed sequential evaluation.

## 10. Diagnosis of ccTGA

The timing of clinical presentation of a ccTGA patient depends predominantly on the type and severity of the associated anomalies. Most patients presenting as young adults have either mild or no associated congenital cardiovascular defects [27]. Only 1–10% of individuals with ccTGA have no associated defects [39,40]. Their life expectancy is limited by the onset of systemic (morphologically right) ventricle failure in their 40s or 50s.

The diagnosis can be accidental, based on the results of chest radiography or electrocardiography performed for other reasons. It can also be established later in childhood, or in an adult patient presenting with complete heart block or cardiac failure, even though systemic right ventricular failure without any associated cardiac defects is rarely observed.

### 10.1. Heart Auscultation

In a physical examination, a significant clinical feature of ccTGA detectable on auscultation is a loud single second heart tone, which is often palpable on the left sternal border because of the anteriorly positioned aortic valve. The second tone is formed by the aortic component. The closure of the posteriorly displaced pulmonary valve is usually inaudible. The auscultatory symptoms change depending on the concomitant anomalies. A murmur will be present in the area of the middle or lower sternal border if there is an associated ventricular septal defect (VSD), pulmonary stenosis (PS), or tricuspid regurgitation (TR). Bradycardia related to high-degree atrioventricular block is a frequent symptom [27].

### 10.2. Electrocardiogram (ECG) in ccTGA

The electrocardiogram demonstrates Q waves in the inferior leads (II, III, and AVF), as well as V1 and V2 precordial leads over the right ventricle, and the absence of Q waves in V5 and V6 leads over the left ventricle, as typically observed in ccTGA. Since the right and left conduction bundles relate anatomically to the respective ventricles, the direction of septal activation is opposite, namely from right to left [8]. This produces a mirror image pattern in the precordial leads, leading to a reversal of the normal QRS morphology. The P-waves and QRS axis may also vary depending on the specific atrial arrangement and location of the AV node and proximal conducting bundles [41]. An atrioventricular block of varying severity may appear. In approx. one-third of cases, atrioventricular conduction disorders are found, usually in the form of block II, IIIº, or complete heart block. Occasionally, atrial arrhythmias and WPW syndrome are present. Concomitant anomalies alter the ECG depending on the type and degree of hemodynamic disorders [42].

### 10.3. Chest Radiography (Chest X-ray)

The abnormalities in chest X-ray images are due to dextrocardia or abnormal cardiac silhouette as a result of L-posed aorta. The X-ray shows a characteristic “sloping shoulder” symptom in the medial mediastinum, which is due to the left-sided position of the aorta forming the upper left outline of the heart. The so-called “humped appearance” of the left heart border is common, but not always present. The enhancement of the shadow of the ascending aorta on the left outline of the heart occurs mainly in individuals with concomitant intracardiac blood leakage and right–left blood leakage. Variations in the heart shape can be found, depending on the presence and severity of the associated anomalies, as well as on the degree of cardiac dysfunction [15,43]. In the cases of a large VSD, an enhanced pulmonary vascular pattern and an enlargement of the heart silhouette can be seen. The pulmonary vascular pattern depends on the concomitant defects, usually narrow vascular pedicle, often dextroversion. Chest radiography is usually normal in the case of an isolated defect.

### 10.4. Echocardiography (Cardiac Ultrasound)

Transthoracic echocardiography (TTE) remains the primary diagnostic tool in ccTGA. This examination allows one to determine the mismatch between the atria and ventricles and the ventricles and large vessels, as well as to assess the location, size, and number of interventricular defects, tricuspid valve anomalies, and the gradient through the outflow tract from the left ventricle. Assessment of the RV function is crucial. Echocardiographic assessment might be challenging, particularly due to the complex morphologic features of the RV cavity, including rich endocardial trabeculations. Since the right ventricle (also in the subaortic position) does not have an axis of symmetry in contrast to the left ventricle, the mathematical assumptions used to assess the volume and ejection fraction of the left ventricle cannot be adopted in echocardiography.

The sRV function is assessed qualitatively (preserved, mildly, moderately, or significantly impaired systolic function) and quantitatively using reliable echocardiographic parameters, designed to assess the systolic function of the right ventricle in the subpulmonary position—geometric, i.e., fractional area change (FAC), and non-geometric, i.e., tricuspid annulus plane systolic excursion (TAPSE), right ventricular efficiency index (Tei), myocardial performance index, (MPI), and tissue Doppler imaging (TDI) parameters (systolic velocity of the lateral part of the tricuspid annulus—S’ wave).

No set of echocardiographic parameters dedicated to the assessment of sRV which would define the severity of its dysfunction has been established to date. Interpretation of the results may also be difficult due to the different characteristics of the sRV fibers (lower number of longitudinal fibers) compared to the subpulmonary chamber. As a result, the obtained values of individual echocardiographic parameters should not be referred to the range of normal values established for the right subpulmonary ventricle.

Among the novel quantitative echocardiographic techniques, the determination of the regional myocardial strain rate, initially performed using the Doppler technique and currently with a new method that analyzes movement by tracking speckles (natural acoustic markers)—speckle tracking echocardiography (STE) on a two-dimensional (2D) ultrasound image. STE used in the examination of patients with arterial trunk transposition after Mustard/Senning corrective procedures has demonstrated that the global longitudinal strain (GLS) of the sRV best correlates with its ejection fraction evaluated by cardiac magnetic resonance (CMR) [44,45,46]. Recent studies of patients with ccTGA highlight the benefits of assessment of the longitudinal functional parameters, and report the global longitudinal strain to be a highly sensitive marker of RV dysfunction in adult patients with an RV ejection fraction < 45% [46,47,48]. The assessment of myocardial strain also has a prognostic value in this group of patients [49,50].

Three-dimensional (3D) echocardiographic imaging also allows for quantitative evaluation of the sRV. However, this method is subject to the error of underestimating the volume of the chamber and depends on the quality of the acoustic window. Currently, there is also no commercially available software dedicated to sRV assessment. Echocardiography, in addition to sRV function, also shows concomitant defects, especially tricuspid regurgitation (systemic atrioventricular valve). Unfortunately, echocardiographic assessment is limited by an insufficient acoustic window (caused by mesocardia) in a significant proportion of patients with ccTGA.

### 10.5. Cardiac Magnetic Resonance (CMR)

Cardiac magnetic resonance (CMR) is regarded as the gold standard imaging modality. In addition to excellent imaging quality for vessels emerging from the heart, it can provide excellent anatomic detailing, including measurements of ventricular volumes and quantification of shunt and valvar regurgitation. Despite their complex morphology, MR images in the short axis make it possible to precisely calculate the systemic right ventricular (sRV) ejection fraction based on systolic and diastolic dimensions [15,51]. CMR is useful for studying complex LVOTO and the feasibility of treating difficult VSD. Unfortunately, this test is expensive and not commonly available, and a significant group of patients with ccTGA have contraindications to its performance (mainly due to arrhythmia and third-degree atrioventricular block, the need for constant pacing-implanted pacemakers not compatible with CMR, and pregnancy).

### 10.6. Computed Tomography in ccTGA

In addition to data on the morphology and function of the sRV, computed tomography provides additional information on anomalies and narrowing of coronary vessels, but it involves the exposure of patients to ionizing radiation and contrast agents.

### 10.7. Cardiac Catheterization

Cardiac catheterization is indicated when it is necessary to precisely determine the hemodynamics and anatomy of pulmonary stenosis and the degree of pulmonary vascular leakage and pulmonary vascular resistance before surgery for the preoperative evaluation of the defect. The assessment of pulmonary vascular resistance is necessary in older patients without any LVOTO [52]. Selective coronary arteriography is also important in view of potential morphological abnormalities and acquired lesions, which can be crucial for medical management and planning of surgical treatment [15]. The indications for cardiac catheterization are currently limited, since better assessment of anatomy, ventricular function, and the severity of atrioventricular valvar regurgitation with echocardiography and magnetic resonance imaging is currently available. The estimated risk of atrioventricular block during cardiac catheterization amounts to 5–10%.

### 10.8. Cardiovascular Three-Dimensional (3D) Printing

Novel technologies such as cardiovascular three-dimensional (3D) printing currently play an important role in preoperative evaluation and planning the surgical strategy for complex CHDs [53,54,55]. The aim of 3D printing or models is mainly a better analysis of complex anatomies to optimize surgical repair or intervention planning [53,55]. Three-dimensional modeling and printing technologies are reliable and appropriate for the analysis of subvalvular ccTGA. Three-dimensional models are complementary with echocardiography that is currently the only modality allowing an adequate description of the atrioventricular valves and their subvalvular apparatus.

### 10.9. Ergospirometry Test

The ergospirometry test is an exercise test that is the basis for the assessment of cardiopulmonary function and the extent of clinical deterioration, as well as the rate of disease progression [15]. Adult patients with ccTGA have diminished aerobic capacity [49]. The aerobic capacity in these patients was found to range from 30 to 50% of the expected value achieved by healthy people [56], which is due to the lack of increase in ventricular ejection fraction during exercise and inadequate chronotropic response. Decreased heart rate, forced expiratory volume in one second (FEV1), forced vital capacity, and systolic blood pressure compared to the predicted values may contribute to the reduced maximal oxygen uptake (VO(2)max) observed in patients with ccTGA. Additionally, a limited increase in systolic right ventricular ejection fraction and a decrease in pulmonary left ventricle contractility are suggestive of a dysfunction of both ventricles [56].

There was also a significant correlation observed between the predicted peak oxygen uptake (%pVO2) and RV ejection fraction by CMR and the Tei index obtained by echocardiography [57]. A recent study in adults with a systemic RV demonstrated peak oxygen uptake, peak heart rate, and percentage of maximal heart rate on exertion to be significantly lower compared to a control group. In that study, reduced exercise capacity was associated with impaired systemic RV function, severe TR, and chronotropic incompetence [58].

The hemodynamic, clinical, and radiological picture of ccTGA associated with other cardiac anomalies varies depending on the type and degree of the concomitant defect. CMR, echocardiograms, and catheterization are the most accurate and definitive procedures which reveal the precise morphological nature of the anomaly and also help to assess the degree of tricuspid valve regurgitation, ventricular function, and effects of various anomalies on the hemodynamics of this defect.

## 11. Cardiac Biomarkers. Clinical Significance of BNP and NT-proBNP Concentrations in Patients with ccTGA

Cardiac biomarkers, advanced imaging, and/or hemodynamic assessment may be helpful in differentiating the ccTGA patients who will do well for decades without the necessity of any intervention from those who will not.

The number of patients with ccTGA surviving to old age is increasing [35,36]. Therefore, in order to characterize the systemic right ventricle (sRV) in terms of the clinical profile, commercially available software dedicated to its assessment is sought. Neurohormonal activation has been demonstrated to be related to symptom severity and systemic ventricular dysfunction in patients with congenital heart disease [59], and natriuretic peptides are considered to be potentially the best cardiac biomarkers in the assessment of sRV function and long-term prognosis in ccTGA [60,61,62,63].

B-type natriuretic peptide (BNP) and its N-terminal precursor (NT-proBNP) are released from ventricular cardiomyocytes in response to pressure/volume overload or hypoxia, and are clinically useful for ccTGA screening in the neonatal stage [64].

Increased levels of natriuretic peptides are associated with worse clinical outcomes, indicate more advanced disease, and are associated with a risk of sudden death [63,64,65,66], whereas decreases in NT-proBNP levels correlate with improvements in heart failure condition [67]. However, the results of research in this area remain inconclusive [68,69]. This is probably due to the dependence of natriuretic peptide concentrations on volume changes in the circulatory system, the age and sex of the patient, and the treatment used [64,66,67].

In addition, due to the extreme rarity of the disease [60,70,71], it is difficult to assess the concentration of natriuretic peptides and to interpret the results obtained in patients with ccTGA. As NT-proBNP is a significant prognostic factor for adverse cardiac outcomes in patients with sRV [63,72] and correlates with echocardiography-derived sRV function parameters, its application in elderly ccTGA patients facilitates diagnosis and therapy.

## 12. Pregnancy

In clinical practice, pregnancy is of particular importance, as it leads to physiological changes in the cardiovascular system that modify the hemodynamics of ccTGA. These include an increase in circulating blood volume and cardiac output accompanied by a decrease in peripheral resistance and blood pressure. Considering the physiological changes occurring during pregnancy, the risk for maternal cardiac complications increases significantly in patients with RV dysfunction and/or more than moderate TR present preceding their pregnancy. On the other hand, blood pressure values increase during labor, and cardiac output increases rapidly (by 50% during labor pains, reaching an 80% increase in the early postpartum period). Taking into account the impact of the physiological adaptation of the cardiovascular system to pregnancy, women with ccTGA are classified as maternal cardiovascular risk class III according to the WHO. This means a high risk of complications, and frequent check-ups (every 1–2 months) by both a cardiologist and an obstetrician are recommended [73]. Two studies reported the histories of 32 patients aged 18 to 40 years who had had 65 pregnancies in order to define maternal and neonatal outcomes [73,74]. Despite the small sample size, pregnancy was successful in most of these patients. However, a few of them developed supraventricular arrhythmias and RV dysfunction. Miscarriages and elective termination of pregnancy were uncommon, and CHD in their offspring was rare. Wissocque et al. have reported an interesting and remarkable case of ccTGA diagnosed at the age of 70 years [75]. Of note, during her lifetime (92 years), this patient had undergone 10 pregnancies without heart failure, and indeed without a diagnosis. Preconception counseling is important and prenatal care should be provided at a tertiary center.

## 13. Surgical Treatment Strategies for ccTGA

### 13.1. Indications for Surgical Intervention

**Figure 3:**
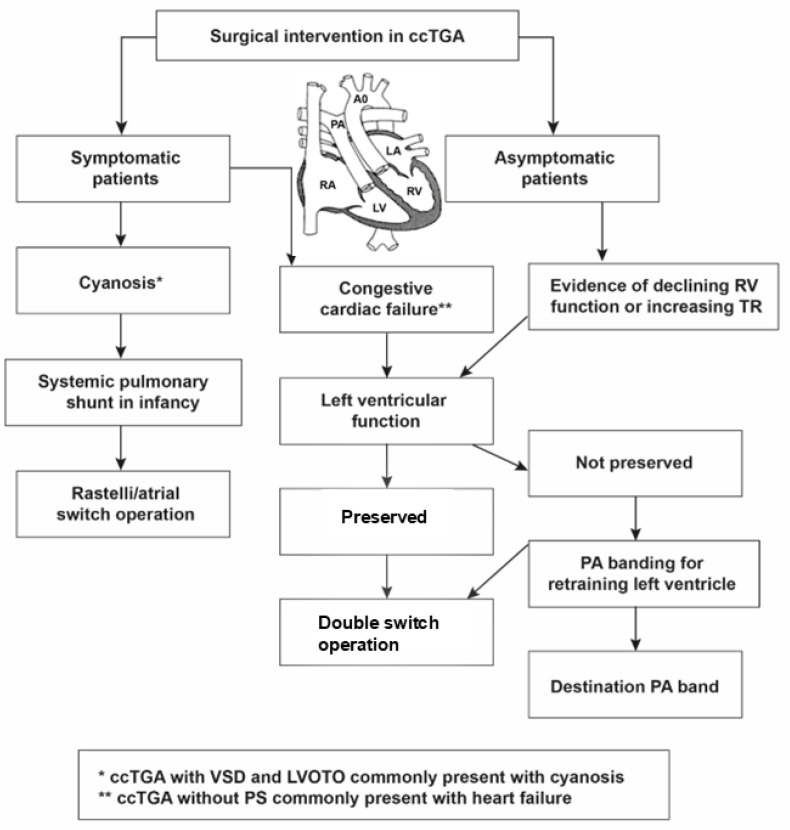
Indications for surgical intervention in ccTGA. CcTGA: congenitally corrected transposition of the great arteries; RV: right ventricle; PA: pulmonary artery. This figure was reproduced with permission from Kumar [29], under the terms of the Creative Commons Attribution License (CC BY).

In ccTGA, indications for surgical intervention typically depend on the physiological consequences of the associated defects. Surgical intervention is reserved mainly for symptomatic patients as well as asymptomatic ones with evidence of declining RV function and worsening tricuspid regurgitation (Figure 3). In asymptomatic patients, even if there are structural defects, surgical interventions are discouraged due to long-term issues, the risk of heart block, and the subsequent possibility of the implantation of a graft of appropriate size [76]. Patients with ccTGA diagnosis established in utero were reported to have a 46% risk of surgery by 32 months of age [77].

### 13.2. Physiological Correction Strategy for ccTGA

The surgical treatment of ccTGA was initially limited to the necessary palliative procedures or elimination of accompanying defects. In this way, a state of physiological correction was achieved, from which the name of the defect (corrected transposition of the great arteries) comes [78,79]. Traditional, classic, or physiological operations on ccTGA involve correcting the septal defects or LVOTO or left atrioventricular valve regurgitation without correction of atrioventricular and ventriculoarterial discordance [80]. The physiological approach may be useful in certain case settings, such as older patients with well-preserved RVs and tricuspid valves who may be high-risk candidates for anatomical repair [81]. Unoperated ccTGA patients are often misdiagnosed in adulthood, and despite symptomatic systemic atrioventricular valve regurgitation and significant RV dysfunction, their referral for treatment is late [79].

Clinical manifestations, which may be present from birth, determine the type of palliative procedure performed at the first stage of treatment (systemic–pulmonary anastomosis, pulmonary arterial trunk banding).

Systemic–pulmonary anastomosis is performed in the case of pulmonary valve atresia or extreme pulmonary stenosis (most often the modified Blalock–Taussig–Thomas shunt), which allows patients to reach the age and body weight appropriate for corrective surgery of the anatomical defect to be performed.

Narrowing of the pulmonary artery (banding) is performed in patients with large VSD to prevent symptoms of circulatory failure and to prevent the development of pulmonary hypertension [76]. The band is tightened under the control of invasive pulmonary artery pressure measurement and transesophageal echocardiography monitoring the function of the heart, so as to obtain the pressure behind the band equal to approx. one-third of the systemic pressure [82,83].

VSD closure: VSD, usually of the malalignment type, causes the pulmonary valve to lie above the interventricular septum, partially arising from the anatomical right ventricle. Because the topography of the conduction system is altered in ccTGA (see Section 8), the risk of blocking occurs when the VSD is closed from the aortic valve [84]. However, this is not a very useful approach in children in early infancy, due to the small size of the valve ring. From the right atrial approach, through the mitral valve, single mattress sutures are used to close the upper and anterior circumference of the defect, with a pad placed approx. 4 mm from the edge, on the left surface of the septum, i.e., within the anatomically right ventricle. These sutures are applied through the VSD. The remaining part of the circumference is closed with a continuous suture, after passing to the right-sided surface of the septum, from the right-located, morphologically left ventricle as described by Leval et al. [80].

In the cases with stenosis in the outflow tract to the lungs, the obstruction usually exists at several levels (fibromuscular tissue, additional tissue associated with the mitral valve, subvalvular tunnel stenosis, valvular stenosis). Complete resection of subvalvular stenosis is hindered by the presence of the subpulmonary cone and the course of conductive tissue. The risk of the occurrence of blocking in cases of resection in this area is very high. The LVOTO can be bypassed by the implantation of a conduit with a valve (homograft, xenograft) between the left ventricle and the pulmonary artery trunk. The left ventricular incision is made in such a way as to avoid damage to the coronary vessels and papillary muscles as well as to the conduction system. VSD is closed by the mitral valve (following the aforementioned rules) or the aortic valve, and sutures are placed on the left side of the septum (the morphologically right ventricle), or by the incised left ventricle. Due to the risk of conduction block, epicardial electrodes are routinely sutured.

In highly selected cases, the physiologic surgical approach may still be beneficial. Patients with good right ventricular and tricuspid valve function, balanced ventricles, and favorable septation anatomy may be eligible, especially if there is a relative contraindication to anatomic repair. In some situations, physiological repair may be the best option in patients with poor mitral valve function, coronary anomalies, small atrium, dextrocardia, or ventricular septal defect features which may complicate anatomic repairs [81,85].

### 13.3. Anatomical Correction Strategy for ccTGA

**Figure 4:**
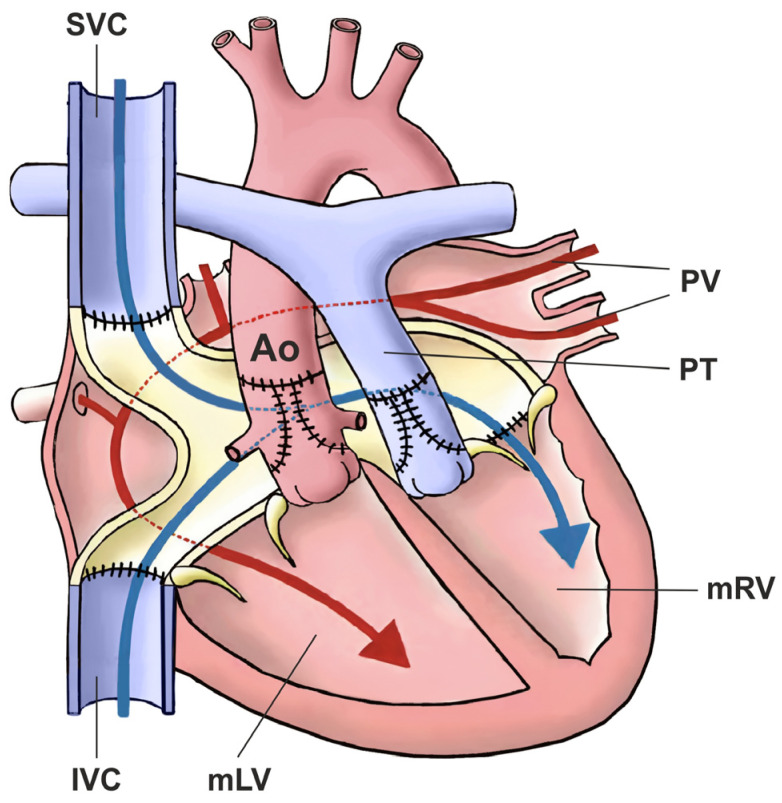
Schematic representation of anatomic repair in the form of a double-switch operation by restoring flow in the normal arrangement. The figure shows the steps involved in the so-called double-switch procedure. Ao: aorta; IVC: inferior vena cava; SVC: superior vena cava; PV: pulmonary veins; PT: pulmonary trunk, mRV: morphologically right ventricle; mLV: morphologically left ventricle.

The ccTGA anatomical correction strategy was first described by Michal Ilbawi and colleagues [86]. The Ilbawi method involves the correction of atrioventricular and ventriculoarterial discordance in the form of a double-switch operation (Figure 4) [86].

Anatomic repair is not a single surgical technique. It is rather a combination of repairs that accomplish the aims to restore blood flow paths through the “normal” sequence of anatomic structures, specifically with the systemic venous drainage to the mRA, then from the mRV to the pulmonary circulation and from the pulmonary venous drainage to the mLA, and then through the mLV to the systemic circulation.

It is clearly the preferable approach for patients with good mitral valve function, balanced ventricles, a septable heart, and reasonable coronary anatomy. Impaired right ventricular function or tricuspid valve function would be the reasons to choose anatomic rather than physiologic repair, as an unfavorable outcome of the latter can almost always be expected in such cases [81].

**Figure 5:**
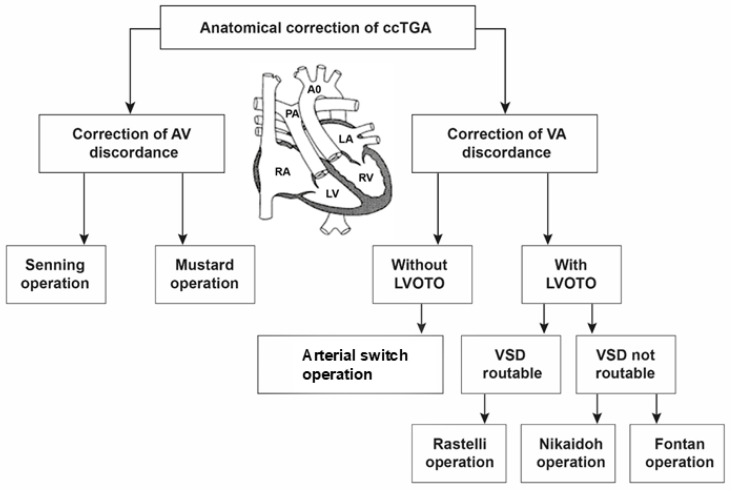
Algorithm for anatomical correction of ccTGA. ccTGA: congenitally corrected transposition of the great arteries; LVOTO: left ventricular outflow tract obstruction; VSD: ventricular septal defect. This figure was reproduced with permission from Kumar [29], under the terms of the Creative Commons Attribution License (CC BY).

Different types of anatomic repair have been proposed to be performed with the aim of correcting double discordance. They combine an atrial stage switch (Senning or Mustard procedure) together with either the Jatene arterial switch operation (ASO) as the double-switch operation or, in cases of relevant left ventricular outflow tract obstruction (LVOTO) and ventricular septal defect (VSD), as a Rastelli procedure, involving intra-ventricular rerouting [3]. Variations of aortic root translocations such as the Nikaidoh or the half-turned truncal switch/en bloc rotation procedures have been introduced more recently [29,78] (Figure 5). The surgical techniques employed and their performance have been described in Part II of our work [87]. One-and-a-half (1.5) ventricular correction for partial anatomic correction has also emerged as a promising choice in recent years [88]. The strategy composes a Glenn operation to unload the RV, hemi-Mustard at the atrial level, and a Rastelli/arterial switch operation (ASO) at the ventricular level. Since the introduction of the Ilbawi procedure, there is more and more convincing evidence in favor of the anatomical correction of this defect.

Several studies have proposed various selection criteria for the evaluation of cc-TGA patients’ eligibility for anatomic repair. The suggested criteria mostly focus on LV pressure load and muscle mass. However, their appropriateness has not been broadly verified [89,90]. The importance of the accurate selection of appropriate patients, optimal timing, and surgical technique for a favorable outcome after anatomic correction is emphasized. The proposed criteria to assess the eligibility of cc-TGA patients for anatomic repair primarily include a patient age ≤ 15 years, LV mass index ≥ 45–50 g/m^2^, LV mass/volume ratio > 0.9–1.5, and systolic LV-to-right ventricular pressure ratio LV/RV > 70–90% [89,90,91,92,93].

The concept of the complete correction of the defect using the technique of physiological correction and anatomical correction emerged as a result of a distant observation of the increase in symptoms of failure of the right ventricle working as a systemic ventricle and the tricuspid valve as a systemic valve. Both the right ventricle and the tricuspid valve are not designed to work under conditions of much higher load in the systemic circulation. Performing double-switch surgery even in a group of patients without concomitant heart defects would eliminate the problem of right ventricular insufficiency and tricuspid regurgitation, which worsens with age.

Techniques for the correction of atrioventricular discordance include the Senning and Mustard operations. However, the correction of ventriculoarterial discordance depends on the presence of LVOTO and the location of VSD (Figure 5).

Using the Senning or Mustard technique, the inflow from the systemic veins is directed to the tricuspid valve, and the outflow from the pulmonary veins to the mitral valve. In the ventricular segment, the defect in the interventricular septum is closed, and at the level of the great arterial trunks, arterial switch surgery is performed. As a result, the left ventricle is incorporated as a blood-pumping chamber in the systemic circulation and the right ventricle works in the pulmonary circulation.

Pulmonary artery stenosis is also used in patients who are eligible for future anatomical correction (atrial switch + arterial switch or atrial switch + Rastelli), in older patients who have previously undergone physiological correction, or who have ccTGA without concomitant defects, and it is necessary to train the left ventricle before anatomical correction. In some cases with unfavorable intracardiac anatomy, pulmonary artery banding can be considered a definitive palliative treatment, in order to achieve balanced pulmonary and systemic circulation, as in cases of concomitant pulmonary stenosis, providing natural banding [94]. A pulmonary artery band is also an effective palliative procedure (“destination band”) in older patients who cannot undergo LV training [83].

A prerequisite for the anatomical correction of ccTGA is a conditioned LV. In order to prepare (train) the left ventricle for the anatomical correction of the defect, the band is tightened so that the pressure in the pulmonary artery is 80–100% of the systemic pressure. In some cases, the patient’s condition requires step-by-step tightening of the band. The left ventricle slowly adapts to sudden changes in afterload and intracardiac leakage and may not tolerate too-tight banding initially [93,95].

A better adaptation of the ventricle to the new hemodynamic conditions is provided by gradual, step-by-step tightening of the band with control of the chamber function and its mass gain by means of magnetic resonance imaging at 6–8-month intervals [96,97].

**Figure 6:**
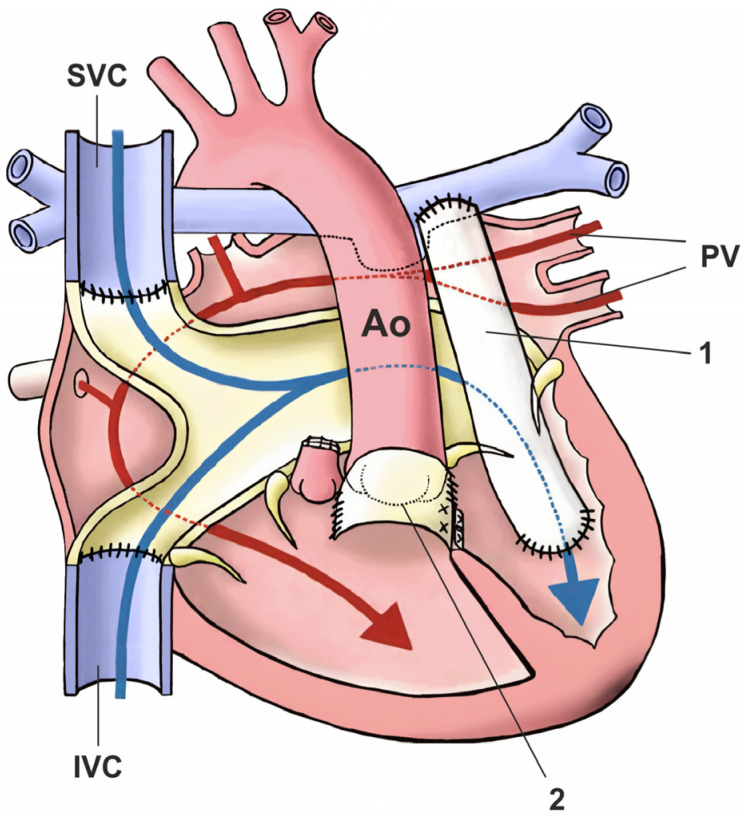
Schematic representation of the Rastelli–Senning operation. The figure shows the final result after an atrial redirection procedure combined with intra-ventricular rerouting of the ventricular septal defect to the aorta, and the placement of a conduit from the morphologically right ventricle to the pulmonary arteries. Ao: aorta; IVC: inferior vena cava; SVC: superior vena cava; PV: pulmonary veins; 1: conduit from morphologically right ventricle to pulmonary arteries; 2: interventricular tunnel from morphologically left ventricle to aorta.

In the cases accompanied by left ventricular outflow tract stenosis or pulmonary valve anomaly, a combination of the physiological correction technique (Senning or Mus-tard) with the Rastelli technique is used (Figure 6). In qualification for the surgery, it is important to determine the function and morphology of the atrioventricular valves as well as the type and location of the defect. Muscular or periapical VSD, tricuspid dysplasia or stenosis, and straddling and/or overriding of one of the valves may exclude the surgery. In the atrial segment, the Senning or Mustard operation is performed, the VSD is closed so that the inflow from the left ventricle is directed to the aorta (usually the defect requires surgical widening), the pulmonary artery is sutured, and a conduit is implanted between the right ventricle and the pulmonary artery trunk.

**Figure 7:**
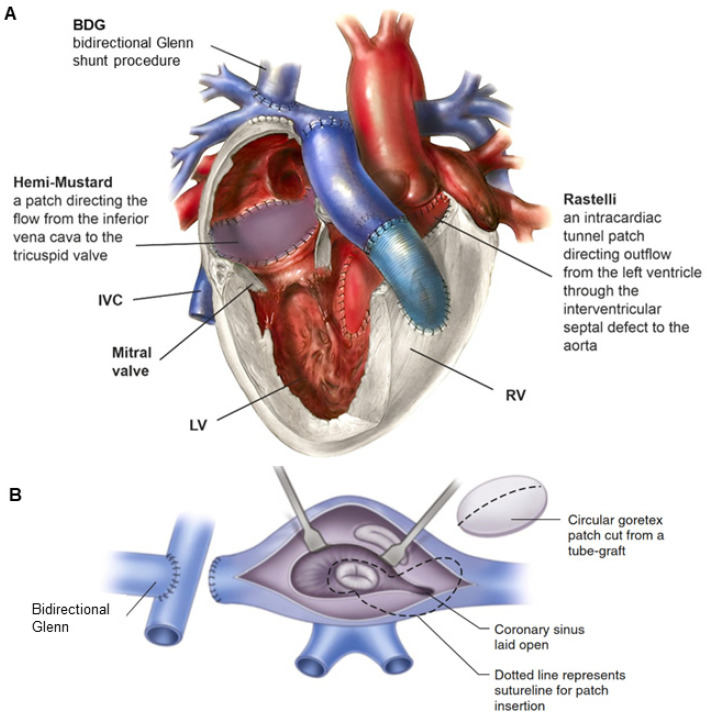
Schematic representation of the hemi-Mustard–Rastelli–Glenn operation. (**A**) Diagram of hemi-Mustard/bidirectional Glenn (BDG) operation with the Rastelli–atrial switch procedure in a dextrorotated heart. BDG: bidirectional Glenn shunt; IVC: inferior vena cava; LV: left ventricle; RV: right ventricle. (**B**) The “Hemi-Mustard” technique. A bidirectional Glenn shunt has been performed and the atrial septum has been excised. A circular patch of Goretex^®^ is used to baffle the IVC through to the tricuspid valve. The coronary sinus has been laid open to give extra volume to the pathway. Figure (**A**) was modified and adapted from Malhorta et al. [88].

In the cases where tricuspid valve anomalies coexist, varying degrees of right ventricular hypoplasia are possible. Then, as part of the double-switch operation, a bidirectional Glenn shunt (Glenn operation) is performed as well as the so-called hemi-Mustard operation (only half of the systemic blood, i.e., blood from the inferior vena cava, is diverted to the tricuspid valve inside the atrium), while the superior vena cava is connected directly to the right pulmonary artery via the Glenn operation, resulting in the repair of one and a half ventricles (Figure 7) [88]. This solution is technically simpler, improves right ventricular hemodynamics, reduces the risk of obstruction at the level of systemic veins, prolongs the duration of pulmonary conduit function, and reduces the incidence of postoperative arrhythmias [88].

The best surgical solution for ccTGA remains controversial and can probably be considered dependent on both the anatomical features of the patient and the experience of the cardiac surgeon.

## 14. Fontan Operation for ccTGA

The Fontan operation, which is performed mainly in patients with univentricular congenital heart disease, is another (albeit less frequently) employed option for ccTGA [81,98].

The Fontan operation is another way to deal with the general problem of AV and VA discordance, committing both RV and LV to the systemic circuit. The general concept of a Fontan operation is to separate the pulmonary circulation from the systemic circulation and achieve proper oxygenation of the body, resulting in the creation of a Fontan circulation [99,100]. The main systemic veins are connected to the pulmonary artery. As a result, a single ventricle secures the systemic circulation, but there is no subpulmonary pump pumping blood into the pulmonary circulation, the blood flow to which is passive. The procedure eliminates cyanosis and reduces the load on the ventricle, but due to its very high dependence on systemic venous pressure, it reduces the cardiac output capacity and increases the adaptation to exercise [101].

The Fontan operation is used in situations where ccTGA is present with VSD and LVOTO and it is impossible to perform the Rastelli procedure because of unfavorable anatomy as a result of dextrocardia, multiple or remote VSD, coronary anomalies, straddling atrioventricular valves, or ventricular hypoplasia [81,99]. The application of the Fontan operation may be limited by tricuspid insufficiency as the valve is still subjected to systemic pressures [102]. The use of the Fontan in ccTGA is most accepted for patients with a large VSD felt to be unsuitable (or at high risk) for ventricular septation.

The results confirming a significant improvement in treatment when switching to a strategy using a bidirectional cavopulmonary shunt as a staging procedure are of key importance for the usefulness of the Fontan strategy in ccTGA. Nowadays, Fontan operative risk approaches zero in many experienced centers [103].

There are limited specific data regarding Fontan outcomes in ccTGA compared to those concerning other repairs. The patients subjected to a Fontan operation, some followed for as long as 20 years, performed at least as well as patients undergoing anatomic repair in terms of late survival and freedom from reoperation [102].

The advantages of the Fontan operation include low operative risk and superior early surgical outcomes, but this technique carries with it the inherent features of chronic systemic venous hypertension, low cardiac output, and liver fibrosis, which could translate into poor quality of life and worse long-term outcomes [104,105].

## 15. Postoperative Period and Treatment Results

Procedures limited to physiological correction are associated with low mortality [78]. The predominant problems in the early postoperative period are arrhythmias and conduction disorders I, II, or III° block). They can occur at any stage of treatment, including the initial palliative procedures. In some cases, they are unrelated to the closure of VSD (spontaneous atrioventricular block). Other problems reported after physiological correction include residual VSD, left ventricular outflow tract stenosis, and tricuspid regurgitation [106].

The occurrence of complete atrioventricular block is an indication for the implantation of a pacemaker. Usually, in children weighing less than 15 kg, the DDD system is implanted epicardially, with access by sternotomy or thoracoscopy. In patients weighing more than 15 kg, the pacemaker can be implanted transvenously.

The most common and serious problem in the distant postoperative period is the dysfunction of the right ventricle subjected to systemic afterload. As a result of overloading, hypertrophy and fibrosis develop. Arrhythmias and conduction disorders contribute to decreased cardiac output, increased end-diastolic volume, and increased stroke volume, which in turn leads to ventricular dilation, increased tricuspid regurgitation, and symptoms of heart failure [16,86].

The results of treatment with the anatomical correction method (double switch) are very good. Perioperative mortality is 0–7% [96,107]. Total atrioventricular block appears in 2–5% of patients, and its incidence increases when VSD enlargement is necessary [88]. Aortic valve regurgitation, which is most likely the result of the previous use of a initial pulmonary artery trunk banding, is also a problem described after anatomical correction [108].

## 16. Cardiac Devices Supporting Circulation

### 16.1. Defibrillators and Stimulators

If advanced second- or third-degree atrioventricular block is present, pacemaker implantation may be necessary. However, late-onset systemic ventricular dysfunction is the most common complication after pacer insertion [109]. Systemic right ventricular dysfunction in ccTGA increases the risk of sudden death and ventricular tachyarrhythmias.

For the time being, it remains unclear whether defibrillators offer survival benefits [110].

### 16.2. Cardiac Resynchronization Therapy (CRT)

Cardiac resynchronization therapy (CRT) is feasible in ccTGA [111,112]. CRT in adults with repaired congenital heart disease is still a challenge. Preliminary data suggest CRT as a potentially effective option in the cases of systemic right ventricular dysfunction that may potentially limit some of the negative pacing-induced effects [112,113,114].

Patients with ccTGA often develop conduction disease and complete atrioventricular (AV) block because of developmental anatomical anomalies. Additionally, AV block often occurs after corrective cardiac surgery, particularly in the context of ventricular septal defect (VSD) closure [19]. There is still a controversy regarding optimum pacing modality in the cases of permanent pacemaker implantation. Although it is possible that pacing-induced ventricular dyssynchrony plays an important role in the functional deterioration of ccTGA patients [115], CRT involving biventricular pacing or His bundle pacing remains challenging in this population, particularly in patients who have undergone surgical anatomical correction (double-switch surgery) when there is typically no direct access to the conduction system or to the distal coronary sinus (CS). The recently introduced left bundle branch area pacing (LBBAP) is a promising alternative to deliver physiological pacing [116,117].

Ventricular assist device placement and heart transplantation are the options to be considered in patients with persistent heart failure refractory to standard medical and surgical management.

### 16.3. Ventricular Assist Device (VAD)

Mechanical ventricular assist devices (VAD) are commonly used to treat severe heart failure, to support or replace the function of the failing heart. These devices, commonly referred to as artificial ventricles, are designed to support the work of the cardiac ventricles: left (LVAD), right (RVAD), or left and right (BiVAD), depending on the patient’s condition.

There are several reports limited to small groups or single patients describing the implantation of a ventricular assist device (VAD) into a systemic RV in ccTGA [118,119,120]. In patients with ccTGA, VADs have two significant advantages over other interim procedures as a bridge to transplantation: effective decompression of the systemic RV and postponing further development of non-cardiac symptoms of heart failure. Moreover, they are independent of TV function. Therefore, their implantation may be beneficial for patients with severe TR. The implantation of microaxial VADs provides compensation for the systemic RV failure, improvement of the patient’s quality of life, and reduction in the incidence of abdominal wound dehiscence; thus, the patient has a better chance for VAD explantation [119,120]. In 2013, Shah et al. reported the largest series of VAD implantations performed in patients with systemic RVs [120].

## 17. Cardiac Transplantation

Heart transplantation is the gold standard in the treatment of terminal heart failure caused by CHD [121]. Orthotopic heart transplantation may be the only option for some ccTGA patients either too old for LV retraining and anatomical repair or presenting poor outcomes after anatomical surgery [122]. Patients with decompensated sRV failure unresponsive to or ineligible for CRT and resistant to medical therapy should be referred to a transplantation center [123]. While it can significantly prolong their life, serious complications are common, including graft rejection and multisystem disease secondary to necessary postoperative management and immunosuppression.

The risk factors for late mortality or transplantation include atrial fibrillation, systemic right ventricular ejection fraction < 40%, and subpulmonary ventricular systolic pressure > 50 mm Hg [124,125]. Technical modifications are necessary to achieve good outcomes [126].

## 18. Summary

ccTGA is a rare cardiovascular anomaly, accounting for less than 1% of all congenital heart defects. Its essence is the AV and ventriculoarterial (VA) discordance, exhibiting discordant atrioventricular and ventriculoarterial connections and the parallel arrangement of the great vessels. The majority of patients have additional anomalies, such as ventricular septal defect (VSD), pulmonary stenosis, tricuspid valve disease, and abnormal position and course of the coronary vessels. Cyanosis and dyspnea are common presenting symptoms, resulting from RV or tricuspid valve function failing at systemic pressure, or from associated anomalies such as LVOTO and VSD. Patients remain asymptomatic for a long time; the symptoms are associated with the presence of additional defects, insufficiency of the morphologically right systemic ventricle, arrhythmia, and anomalies resulting from anatomical abnormalities of the conduction system. Progressive AV block, requiring permanent pacing, is frequent. The natural history of ccTGA varies depending on manifestations in individual patients, on the coexisting malformations and the need for cardiac surgery. Isolated ccTGA may, in favorable scenarios, remain unrecognized for decades. The presence of associated abnormalities is associated with a high health deterioration rate in adult life. Heart failure develops gradually, usually up to the fourth or fifth decade of life, as a result of prolonged pressure and volume overload on the right ventricle trying to meet the systemic demand. However, the number of patients with ccTGA surviving to old age is increasing. The surgical management of ccTGA has evolved from physiological repair addressing only the associated structural defects to an anatomic repair establishing the morphological LV as the systemic ventricle. Although the outcomes have improved with anatomic repair, well-designed multicenter studies in elderly patients with sRV are required to refine the indications for surgical intervention, especially in the absence of associated structural defects. Although clinical presentation and diagnostic criteria have been well established, there is still room for discussion related to clinical and surgical management of ccTGA in adult patients.

## 19. Conclusions

ccTGA is a cardiac anomaly that can be diagnosed as early as in the prenatal period. Life-long follow-up is recommended for the great majority of cases. Most of these patients are recommended to be monitored for life in the ACHD outpatient clinic. Routine Holter monitoring is very important for these patients because of the risk of spontaneous heart block. The application of new quantitative imaging techniques, such as echocardiography, measurement of regional deformations of the heart muscle (strain rate), contrast echocardiography using echogenic microbubbles, and cardiac magnetic resonance imaging enabling the assessment of anatomy and function, has provided valuable diagnostic and prognostic tools. In acute and chronic management of cardiac failure, the aim of structural and surgical interventions in conjunction with up-to-date pharmacologic, ablative, and device-based therapies is palliation and prevention of further decompensation. Mechanical support should be considered as a bridge to transplantation or destination therapy in patients whose heart failure is refractory to medical and other adjunct therapies, such as resynchronization therapy. Physicians managing patients with ccTGA should take a multidisciplinary specialist approach to choose the most appropriate route to pursue and to decide when more advanced treatment options should be implemented.

## Data Availability

Data sharing not applicable.

## Abbreviations

ASO	arterial switch operation
AtrSR	atrial switch repair
AV	atrioventricular
BNP	B-type natriuretic peptide
cc-TGA	congenitally corrected transposition of the great arteries
CD	color Doppler
CHD	congenital heart defect
CM	cardiomyocytes
CMR	cardiac magnetic resonance
CRT	cardiac resynchronization therapy
CS	coronary sinus
CT	computed tomography
DA	ductus arteriosus
D-TGA	dextro transposition of the great arteries
ECG	electrocardiogram
ECHO	echocardiography
FEV1	forced expiratory volume in one second
GLS	global longitudinal deformation
LA	left atrium
LBBAP	left bundle branch area pacing
L-TGA	L-looped transposition of the great arteries
LV	left ventricle
LVOT	left ventricle outflow tract
LVOTO	left ventricle outflow tract obstruction
NT-proBNP	N-terminal pro-B-type natriuretic peptide
PA	pulmonary artery
PAB	pulmonary artery banding
PS	pulmonary stenosis
RA	right atrium
RV	right ventricle
SS	situs solitus
SI	situs inversus
sRV	systemic right ventricle
STE	speckle tracking echocardiography
TR	tricuspid valve regurgitation
TV	tricuspid valve
VAD	ventricular assist devices
VSD	ventricular septal defect
X-ray	radiological examination
